# Seated Virtual Reality-Guided Exercise Improved Gait in a Postoperative Hallux Valgus Case

**DOI:** 10.3390/ijerph182413267

**Published:** 2021-12-16

**Authors:** Masami Nakamoto, Akihiro Kakuda, Toshinori Miyashita, Takashi Kitagawa, Masashi Kitano, Masahiko Hara, Shintarou Kudo

**Affiliations:** 1 Inclusive Medical Science Research Institute, Morinomiya University of Medical Sciences, 1-26-16, Nanko-kita, Suminoe-ku, Osaka 559-8611, Japan; kakuda@morinomiya-u.ac.jp (A.K.); miyashita.osaka@gmail.com (T.M.); 2021dms001@s.morinomiya-u.ac.jp (M.K.); hara@medivr.jp (M.H.); kudo@morinomiya-u.ac.jp (S.K.); 2 Department of Physical Therapy, Morinomiya University of Medical Sciences, 1-26-16, Nanko-kita, Suminoe-ku, Osaka 559-8611, Japan; 3 Graduate School of Health Science, Morinomiya University of Medical Sciences, 1-26-16, Nanko-kita, Suminoe-ku, Osaka 559-8611, Japan; 2020mhs003@s.morinomiya-u.ac.jp; 4 mediVR, Inc., 106-2-3-8, Terauchi, Toyonaka-shi, Osaka 561-0872, Japan

**Keywords:** single-case study, ABA experimental design, virtual reality, hallux valgus, seated rehabilitation

## Abstract

Virtual reality (VR)-guided exercise therapy using mediVR KAGURA has been reported to improve gait function by extending the arm to spatial targets while sitting. We aimed to investigate toe and trunk–pelvic function and plantar sensation during gait in a postoperative patient with hallux valgus. A 60-year-old woman, whose foot deformities had improved 6 months earlier, participated in the study. The exercise therapy interventions were performed twice weekly for 15 min. This study used an A-B-A design: 1-week pre-phase, 3-week intervention phase, and 2-week post-phase. The plantar pressure distribution and thoracic and pelvic displacements during gait were recorded at the end of each phase. The tactile pressure thresholds of the foot were determined before and after each exercise. The maximum force and impulse under the hallux increased after the intervention. The sensory threshold of the hallux was reduced. The amplitude of the thoracic and pelvic displacement was shortened in lateral and extended in the vertical and progressional directions after the intervention. We found that a 3-week VR-guided exercise improved toe function, plantar sensation, and postural adjustment of the trunk and pelvis during gait in a patient who had undergone surgery for hallux valgus, and the effects continued for 2 weeks.

## 1. Introduction

Deformities of the hallux valgus cause chronic pain. This pain disrupts gait, affects the whole body, and reduces the quality of life [1,2]. Conservative treatment for hallux valgus includes exercise therapies, such as strengthening of the hallux abductor, orthotic therapy, and medication. If the pain does not improve with these treatments, surgery is indicated [3]. However, in cases of hallux valgus after correcting the alignment by surgery, gait problems often persist and cause recurrent deformity and pain [4,5]. It is necessary to improve gait in conjunction with local conservative treatment and surgery. In addition, rehabilitation for hallux valgus is often provided at an outpatient clinic, and the effects of exercise therapy on gait should last for several weeks. However, there are a few reports on effective exercise therapy for gait in patients with hallux valgus [6].

mediVR KAGURA^®^ (mediVR, Inc., Osaka, Japan) has been reported to improve walking ability without increasing lower limb muscle strength [7,8]; however, the mechanism remains unclear. Exercise therapy with the use of virtual reality (VR) requires quick arm movements extending to spatial targets. This reaching motion has been shown to improve the movement strategy of the trunk [9] and upper limb function [10]. We hypothesized that the load on the pelvis and lower limbs with trunk movement could improve gait function. Therefore, in this study, we investigated the effects of VR-guided sitting exercise on toe and trunk–pelvic function during gait in a postoperative patient with hallux valgus.

## 2. Materials and Methods

### 2.1. Participant

A 60-year-old woman participated in this study. Her height and weight were 156.5 cm and 58.0 kg, respectively. At the time of participation, 6 months had passed since bilateral surgery for hallux valgus, and the hallux valgus angles were 7.3° on the right and 14.8° on the left. She had been bothered by the hallux valgus since she was in her 50s, and had been using commercially available wide shoes and a nighttime toe separator splint to reduce the pain, which interfered with her daily life. Subsequently, she visited a hospital for the first time and immediately underwent surgery as indicated, 6 months before our intervention. She was hospitalized for three weeks, postoperatively, and underwent standard exercise therapy for hallux valgus, range of motion exercises, and muscle strengthening training for the feet. She was independent in her daily life and returned to work after being discharged from the hospital. There were no other therapeutic interventions in the last 5 months. However, she still experienced pain and unsteadiness during gait. The level of pain at the time of intervention in this study was 5 on the Numerical Rating Scale (NRS).

This study was approved by the Research Ethics Review Committee of Morinomiya University of Medical Sciences (reference number: 2020-006). The purpose of this study was explained to her, and consent was obtained before the study was conducted.

### 2.2. Experimental Design

**Figure 1:**
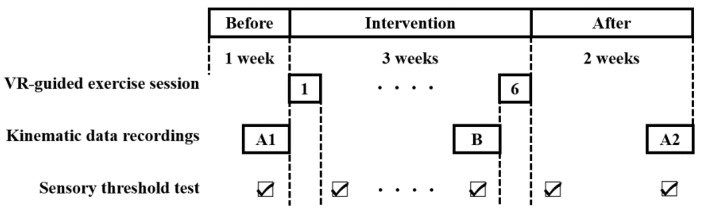
Experimental design. A1 phase, 1 week before intervention; B phase, 3-week intervention (twice-a-week exercise); A2 phase, 2 weeks after the intervention. Kinematic data during gait are recorded at the end of each phase, and the sensory test is conducted before and after each exercise session.

This study was a single case, with an A-B-A design: 1 week before intervention phase (A1), 3-week intervention phase (B), and 2 weeks after the intervention phase (A2) (Figure 1). Each VR-guided exercise session was performed for 15 min, twice a week (six sessions in total). Kinematic data during gait were recorded at the end of each phase, and the sensory threshold of the sole was identified before and after each exercise session. These measurements were conducted during the workday to minimize disruption to daily life.

### 2.3. Intervention

**Figure 2:**
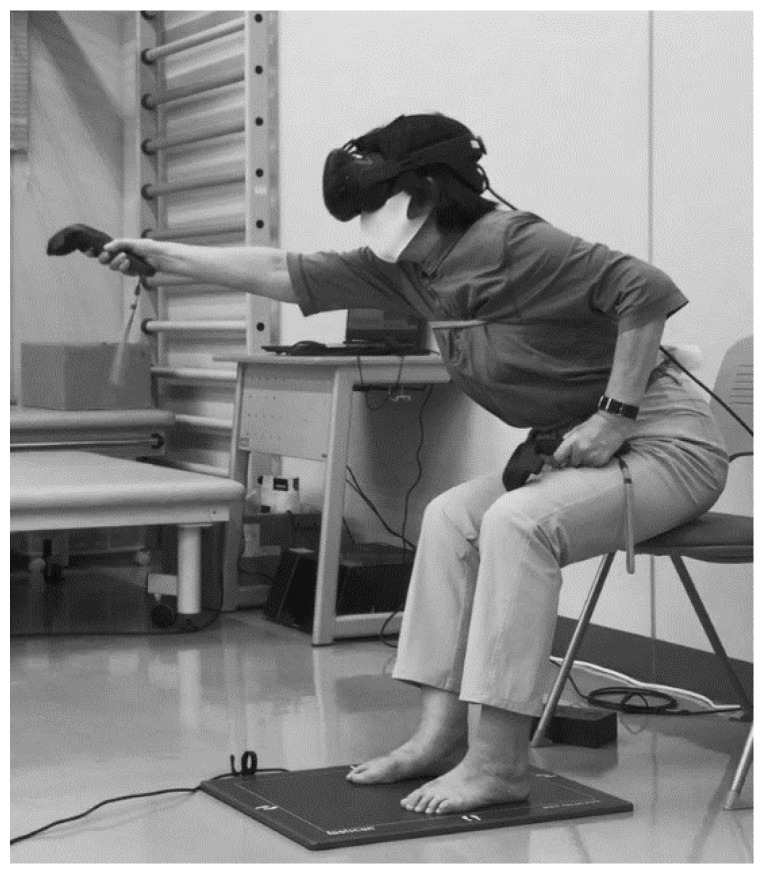
Images of the mediVR KAGURA-guided exercise.

The VR-guided exercise was performed while sitting in a chair with a backrest, wearing a head-mounted display, and holding a controller with both hands (Figure 2). The participant was instructed to, as quickly as possible, catch red or blue falling objects or red or blue fixed targets in a three-dimensional virtual space, with her right or left controller, respectively. The red objects and targets were presented in the right space only, and the blue ones were presented in the left space only to prevent her from moving across the midline. The objects and targets were presented straight ahead and diagonally 45° ahead on each side and at three distances in each direction (at stretched arm length, 90% of maximum reach length, and halfway between), for a total of 12 locations. The first exercise program was configured manually by a skilled therapist to ensure that physical activity was fully induced. The same program was used for the subsequent exercise sessions.

### 2.4. Measurements

Static and dynamic plantar pressure data were recorded using a Footscan^®^ pressure plate (RSscan International, Olen, Belgium; 578 × 418 × 12 mm with 4096 resistive sensors, a data acquisition frequency of 300 Hz, and a pressure range of 1–127 N/cm^2^), which was connected to a computer. The participant was asked to stand for 60 s and walk at a comfortable speed on the plate, which was located at the center of a 10-m walking path. The recording of dynamic plantar pressure was terminated if the patient stepped on the plate three times with her right foot. The data were analyzed using the Scientific Footscan^®^ software (RSscan International, Olen, Belgium). Footprint impressions in the standing position were obtained, and arch width, which was defined as the length of a perpendicular line drawn from the mid-point of the medial border in the arch area to the mid-foot [11], was measured using a ruler. The maximum force and impulse of the vertical force component at the hallux and first metatarsal zones were calculated, and the average value of the three gait cycles was obtained.

During the gait cycle, thoracic and pelvic displacements were recorded using two small sensors with built-in acceleration and angular velocity sensors (MVP-WS2-S, MicroStone Corp., Nagano, Japan), which were connected to a computer. Each sensor was fixed to her back at the seventh thoracic and first sacral vertebrae. The vertical, lateral, and progressional amplitudes of the thoracic and pelvic displacements were calculated from the obtained data, excluding the gait cycle wherein the patient stepped on the platform. The stride-to-stride time variability (STV) was also calculated [12].

Tactile pressure thresholds of the planter under the hallux and first metatarsal head were identified using Semmes–Weinstein monofilaments (Sakai Medical Co., Ltd., Tokyo, Japan), which consisted of 20 filaments of 0.008–300 g. Pressure was applied for approximately 1.5 s until a bend at a right angle was obtained [13]. The force of the filament with three accurate responses was adopted as the threshold for the site.

## 3. Results

**Figure 3:**
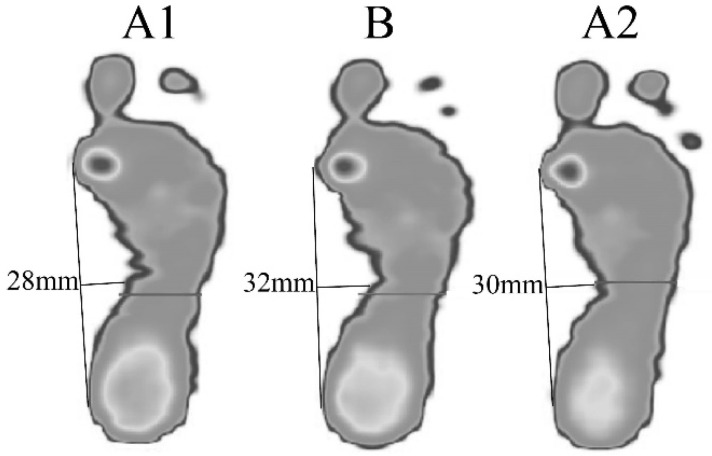
Representations of arch width from the right footprint upon standing in each period.

Arch widths measured from static pressure distribution were 28 mm, 32 mm, and 30 mm in A1, B, and A2 periods, respectively (Figure 3): the NRS for pain was 5, 4, and 5, respectively.

**Figure 4:**
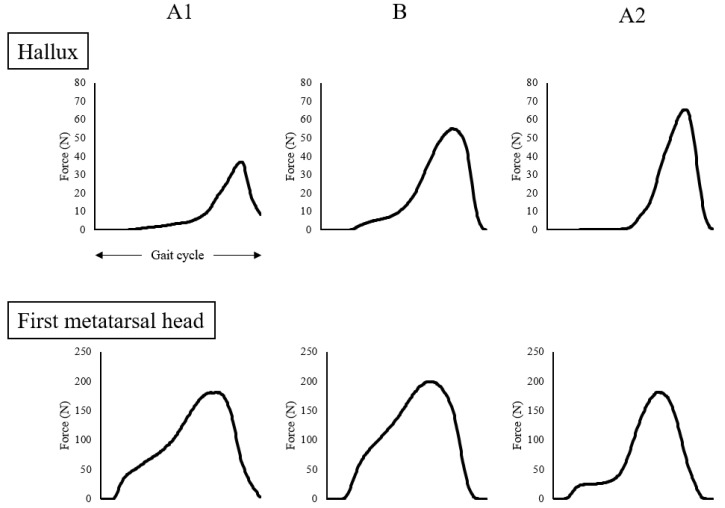
Waveform obtained by adding and averaging the vertical force component of three gait cycles under the hallux and first metatarsal.

**Figure 5:**
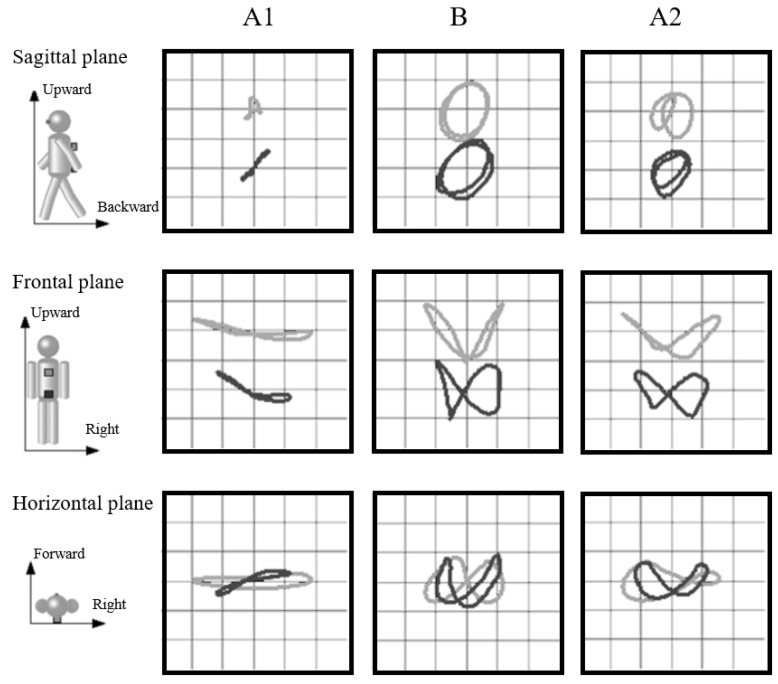
The trajectory of the thoracic and pelvic displacements in the sagittal, frontal, and horizontal planes at the A1–B–A2 phases.

**Figure 6:**
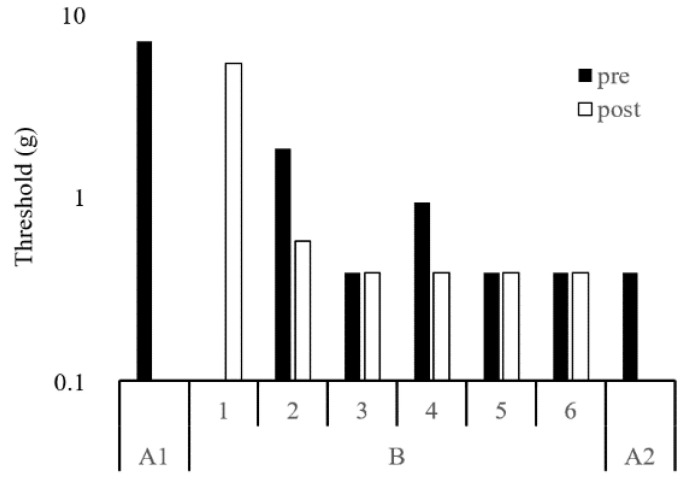
Tactile pressure sensitivity threshold in the right hallux. The vertical axis is logarithmic.

**Figure 7:**
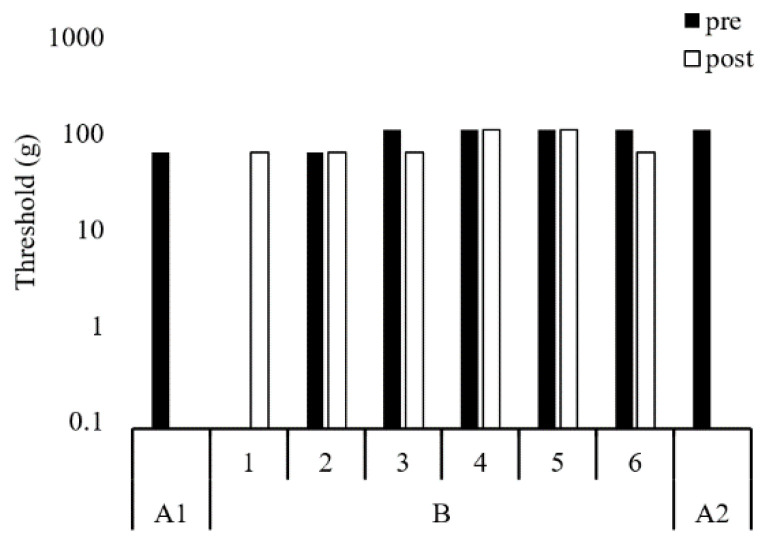
Tactile pressure sensitivity threshold in the right first metatarsal head. The vertical axis is logarithmic.

The results of the kinematic parameters in gait for A1, B, and A2 are shown in Table 1. The maximum force and impulse of the hallux zone during gait increased from A1 to B and A2 (Figure 4). The impulse of the first metatarsal zone decreased from A1 to A2 (Figure 4). The amplitudes of the thoracic and pelvic displacement in the vertical and progressional directions were extended, as were those of thoracic displacement in the lateral direction from A1 to B and A2 (Figure 5). In the frontal plane, the trajectory of these displacements changed into a figure-of-eight (middle row of Figure 5), and the STV decreased from A1 to B and A2 (Table 1). The tactile pressure threshold of the hallux was 8 g in A1 and was lowered to 0.4 g by the third exercise session (Figure 6). There was no significant change in the first metatarsal head, and the threshold was in the range of 60–100 g (Figure 7).

## 4. Discussion

Six months after surgery for hallux valgus, improvements in the load on the hallux and the thoracic and pelvic movements, and stride variability during gait and sensation of the hallux, were observed following VR-guided exercise for 3 weeks; despite this, there were no significant changes in deformity or pain. Furthermore, the effects were maintained after 2 weeks.

Galica et al. [14] found that the maximum force on the hallux of patients with hallux valgus (66.2 ± 10.5 years, 74.5 ± 16.3 kg) was 64.9 ± 41.7 N, which was significantly lower than that in healthy elderly people (64.3 ± 9.7 years, 80.9 ± 17.9 kg, 78.4 ± 39.7 N). In the present case (58.0 kg), the maximum force of the hallux was 39.6 ± 3.3 N before the intervention, which is extremely low, even if the difference in weight is considered. However, after the intervention, the maximum force increased to 57.7 ± 20.1 N, which was approximately the same as that of the elderly without hallux valgus, considering the difference in weight. In contrast, the maximum force on the first metatarsal head was 183.2 ± 42.5 N in the present case, which was higher than that in the study by Galica et al. (123.4 ± 63.0 N) [14]. Although the maximum force on the first metatarsal remained high, the impulse decreased 2 weeks after the intervention. These results suggest that toe function during gait was improved by the intervention. Furthermore, the tactile pressure threshold of the hallux was reduced by the third session and maintained thereafter. In cases of hallux valgus, sensory impairment of the foot is often caused by compression of the dorsomedial cutaneous nerve due to deformity of the metatarsal head and/or injury of the dorsomedial cutaneous nerve after surgery [15]. The median tactile sensory thresholds of the hallux and the first metatarsal head in Japanese healthy young people are 0.4 and 0.7 g, respectively [16], and both are 2.0 g in diabetic patients without apparent peripheral neuropathy [17]. In this case, the deformity was corrected by surgery; however, the threshold was 8 g before the intervention. After the intervention, the threshold was reduced to 0.4 g, which is the same level as that of young people. It is unlikely that structural changes, such as repair of damaged nerves or an increase in receptors, occurred in a short time. In fact, the first metatarsal head, which is the surgical site, had a very high threshold and showed no change in the period. The threshold was probably neurologically lowered because of increased sensory stimulation of the hallux during gait in daily life.

The trunk and pelvis move in the vertical, lateral, and progressional directions during normal gait (gait analysis, second edition) [18]. In the present case, the lateral movement in the trunk was significant, and the vertical and progressional movements in the trunk and pelvis were insignificant before the intervention. After the intervention, the former became shorter, and the latter became larger. It has been reported that the amplitudes of vertical, lateral, and progressive displacements in the thoracic vertebrae are 25–95, 20–60, and 5–30 mm [19], and those in the pelvis are approximately 40, 45, and 26 mm [20], respectively, in elderly people. After the intervention, the amplitudes were almost within these ranges, and the trajectory of the trunk and pelvis changed to form a normal figure-of-eight [21]. The movements required in this study included trunk flexion and rotation and pelvic forward tilt in the sitting position within 90% of the maximum effort. Thus, the exercise was not designed to increase the range of motion of the trunk or pelvis. Supposedly, the changes during gait were observed due to improvements in the postural adjustment of the trunk and pelvis.

STV is an index of fluctuation in gait; a two-fold difference was observed in the measured STV values between those with a history of falls and those without a history of falls [22]. In elderly people without an apparent disease, STV has been reported to increase due to a decline in motor function and psychogenic factors such as anxiety during fall [12]. It has been reported that the mean STV was 3.6 ± 1.8 (1.6–10.4%) in people aged 80–89 years [12] and 2.10 ± 0.94 % in people aged 65–69 years [23]. The STV, in this case, was 15.5%, showing a markedly greater variability than the typical value in the elderly. After 3 weeks of intervention, her STV was 3.8%, which is close to the standard values for elderly people. We could not identify the factors that caused the significant decrease in STV, since this study did not measure lower limb muscle strength or conduct any in-depth interviews about pain, self-efficacy, and anxiety. However, it is possible that because the intervention used was not expected to improve knee extension muscle strength [7], the neurological motor function may have improved, resulting in a significant decrease in STV.

This study suggests that the improvement in walking abilities reported in previous studies using mediVR KAGURA [7,8] is related to the changes in toe function, plantar sensation, and postural adjustment of the trunk and pelvis. As mentioned above, the exercise used in this study is more likely to have improved gait function by influencing sensorimotor function rather than by structural changes, such as increasing range of motion or muscle strength, which would otherwise have been the main purpose in general exercise therapy for hallux valgus [24]. In addition, the spatial target reaching exercise while sitting has mainly aimed to train upper limb function [9,10] or trunk function [25], and a few reports have examined the effects on gait using kinematic data. Furthermore, this exercise is not commonly prescribed to improve gait in hallux valgus cases. Therefore, whether the results observed in this study could also be obtained by performing the exercise by therapists would need to be compared in further studies.

On the contrary, the effects of VR therapy on deformity or pain were not observed in this study. Footwear and orthosis reduced hallux valgus-induced pain [26] and prevented severe deformity [27]. The interventions in this study focused on improving gait function rather than reducing pain or deformity. Therefore, VR therapy in this study should be combined with treatment for pain and prevention of severe deformity.

This study has some limitations. In this study, there was neither improvement nor enhancement of the hallux valgus throughout the intervention period. However, in feet with persistent hallux valgus, incorrect active use of the toes could exacerbate the deformity [4,5]. Thus, the risk of deterioration, as measured by hallux valgus angle and arch height, [24] should have been better documented while conducting interventional studies for such cases with hallux valgus. Additionally, there was an inability to load the hallux and abnormal thoracic and pelvic movements during gait, which improved after the intervention. In this exercise, loading of the pelvis and lower limbs, which is necessary during gait, is repeated while sitting. This may have activated the center of gravity shift and plantar sensation during gait and subsequently improved sensorimotor function. However, these mechanisms cannot be clarified because we did not record neurological data, such as electromyography of the trunk and lower limbs, during exercise therapy and gait. Hence, these issues should be examined in detail in further studies.

## 5. Conclusions

This study found that a 3-week VR-guided exercise improved toe function, plantar sensation, and postural adjustment of the trunk and pelvis during gait in a patient who had undergone surgery for hallux valgus, and the effects continued for 2 weeks. It is necessary to examine whether this effect is evident in all cases of hallux valgus. In the future, the VR-guided exercise in this study may be investigated for application in preventive and therapeutic approaches for hallux valgus before surgery and in early treatment after surgery.

## Figures and Tables

**Table 1:** Serial changes in kinematic parameters during gait.

Kinematic Parameter				A1			B			A2		
**Plantar pressure**												
	Maximum force											
		Hallux	N	39.6	±	3.3	57.7	±	20.1	68.3	±	25.7
		First metatarsal	N	183.2	±	42.5	202.0	±	21.5	183.6	±	17.2
	Impulse											
		Hallux	N·s	7.5	±	1.3	14.6	±	6.9	12.7	±	5.3
		First metatarsal	N·s	74.6	±	32.3	77.8	±	10.1	54.4	±	4.3
**Amplitudes of the thoracic and pelvic displacement**												
	Seventh thoracic vertebra											
		Vertical	mm	14			40			30		
		Lateral	mm	76			50			63		
		Progressional	mm	10			31			26		
	First sacral vertebra											
		Vertical	mm	20			40			31		
		Lateral	mm	47			41			47		
		Progressional	mm	17			35			24		
**Stride-to-stride time variability**			%	15.5			3.8			3.2		
